# Activation of G Protein-Coupled Estrogen Receptor Induces p53 and ADAMTS1 to Inhibit Tumor Growth and Suppress Liver Cancer Metastasis

**DOI:** 10.3390/cancers17162623

**Published:** 2025-08-11

**Authors:** Hee Jung Kwon, Ga Seul Lee, Jeong Hee Moon, Joohee Jung

**Affiliations:** 1 College of Pharmacy, Duksung Women’s University, Seoul 01369, Republic of Korea; rnjs973312@duksung.ac.kr; 2 Core Research Facility & Analysis Center, Korea Research Institute of Bioscience & Biotechnology, Daejeon 34141, Republic of Korea; rktmf1013@kribb.re.kr (G.S.L.); jhdal@kribb.re.kr (J.H.M.)

**Keywords:** liver cancer, GPER, G1, ADAMTS1

## Abstract

**Background/Objectives**: Liver cancer is a common cause of cancer-related deaths among men and women globally. A disintegrin and metalloproteinase with thrombospondin motif 1 (ADAMST1) has been associated with various cancers, including prostate, esophageal, renal, and breast cancers. However, its role in liver cancer remains unclear. The aim of this study was to investigate the relationship between G protein-coupled estrogen (GPER) activation via its agonist, G1, and ADAMTS1 in suppressing liver cancer metastasis. **Methods**: Following preliminary assessment of Hep3B, Huh7, and SK-Hep-1 cells, SK-Hep-1 cells were selected owing to their elevated GPER expression and reduced cell viability. Cells were subjected to flow cytometry, RNA sequencing, and proteomics analyses. We established an SK-Hep-1 xenograft model for in vivo analysis. **Results**: We observed G1-induced G2-M phase cell cycle arrest, increased p53 and p21, and decreased cell cycle-related factors. In vivo, G1 significantly inhibited tumor growth and increased p53 protein expression. ADAMTS1, a metastasis regulator, was significantly upregulated by G1. G1 reduced the proliferating cell nuclear antigen and increased E-cadherin expression in SK-Hep-1 cells and in vivo. Tumor invasion was reduced with G1 and ADAMTS1 expression. In vivo, G1 reduced liver metastasis, increased E-cadherin, and decreased vimentin and proliferating cell nuclear antigen in primary tumor tissues and increased ADAMTS1 at the tumor edge. **Conclusions**: GPER agonists, such as G1, show potential for suppressing liver cancer progression and metastasis.

## Simple Summary

GPER activation by G1 induces p53 and ADAMTS1 expression, contributing to the inhibition of liver cancer metastasis. G1 treatment attenuates SK-Hep-1 cell invasion and suppresses tumor growth in xenograft models. ADAMTS1 upregulation via GPER modulates the extracellular matrix, reducing invasiveness and metastatic potential. Elevated ADAMTS1 expression is associated with improved survival outcomes in male liver cancer patients. These findings highlight GPER agonists as potential therapeutic agents for liver cancer treatment.

## 1. Introduction

Liver cancer is the second leading cause of cancer-related deaths in men and the fifth in women [1]. Liver cancer metastasis is associated with poor prognosis. Men are more susceptible to liver cancer than women, owing to the influence of sex hormones [2]. Specifically, patients with G protein-coupled estrogen receptor (GPER)-positive liver cancer exhibit a longer overall survival (OS) than GPER-negative liver cancer [3]. GPER is expressed in various organs, including the liver, pancreas, and reproductive system, where it modulates different disease-related functions [4]. GPER activation by estrogen and its agonist (G1) directly regulates cell proliferation and migration through the PI3K/AKT and EGFR-MAPK/ERK pathways [5]. In pancreatic cancer, G1 suppressed cell proliferation and tumor growth. Similarly, G1-induced G2/M cell cycle arrest and inhibition of tubulin polymerization suppressed tumor growth in ovarian cancer cells [6,7]. Additionally, GPER-knockout mice experienced increased inflammation and fibrosis, accelerating liver tumor formation [8]. Although GPER enhances migratory phenotypes in breast, lung, and ovarian cancers, contradictory findings exist for triple-negative breast cancer, where GPER activation inhibits migration and invasion [9,10]. These findings suggest that GPER facilitates the suppression of cancer cell proliferation and metastasis in various cancer types.

A disintegrin and metalloproteinase with thrombospondin motif 1(ADAMTS1) is a member of the ADAMTS family, which consists of secreted zinc-dependent metalloproteinases [11]. Unlike membrane-bound ADAM family members, which are involved in cell surface protein shedding and cell–cell interactions, ADAMTS proteins are secreted into the extracellular space and primarily participate in extracellular matrix (ECM) degradation and remodeling [12]. Cancer cells degrade ECM during metastasis and invade surrounding tissues. ADAMTS1, which targets ECM components, plays a vital role in cancer metastasis [11]. Reportedly, ADAMTS1 promotes tumor formation and metastasis in renal and breast cancers. ADAMTS1-L1CAM-EGFR axis drives epithelial–mesenchymal transition (EMT) and lymph node metastasis in oral squamous cell carcinoma [12,13,14]. Studies have shown that ADAMTS1 suppresses lymphangiogenesis and metastasis in esophageal squamous cell carcinoma [15]. ADAMTS1 is associated with hepatic fibrosis suppression [16]; however, the role of ADAMTS1 in liver cancer metastasis remains unclear.

In this study, we aimed to investigate the relationship between GPER activation via G1 and ADAMTS1 in suppressing liver cancer metastasis, proposing a mechanism wherein GPER activation may suppress metastasis through ADAMTS1 regulation.

## 2. Materials and Methods

### 2.1. Cell Culture

Liver cancer cell lines SK-Hep1, Hep3B, and Huh7 were used in this study. SK-Hep1 (KCLB No. 30052), Hep3B (KCLB No. 88064), and Huh7 (KCLB No. 60104) cell lines were purchased from the Korean Cell Line Bank (KCLB, Seoul, Korea). SK-Hep-1 cells were cultured in Dulbecco’s modified Eagle’s medium (GenDEPOT, Barker, TX, USA), Hep3B cells in Eagle’s minimum essential medium (GenDEPOT), and Huh7 cells in RPMI1640 medium (GenDEPOT). All media were supplemented with 10% heat-inactivated fetal bovine serum (FBS; GenDEPOT) and 1% penicillin–streptomycin (GenDEPOT). Cells were cultured at 37 °C in a humidified atmosphere with 5% CO_2_.

### 2.2. Cytotoxicity

SK-Hep-1, Hep3B, and Huh7 cells were seeded at a density of 1 × 10^4^, 8 × 10^3^, and 2 × 10^4^ cells/well, respectively, in 96-well plates. SK-Hep-1, Hep3B, and Huh7 cells were treated with 1, 3, 5, and 10 μM of G1 (Cayman Chemical, Ann Arbor, MI, USA ) and G15 (Cayman Chemical). G1 (Cayman Chemical) and G15 (Cayman Chemical), both dissolved in 0.1% dimethyl sulfoxide (DMSO, Sigma-Aldrich, St. Louis, MO, USA) for 24 h. To measure cell viability, 10 μL of MTT solution (Sigma-Aldrich, 5 mg/mL in phosphate-buffered saline [PBS]) was added into each well, followed by incubation at 37 °C for 3 h. Thereafter, the supernatant was removed, and 100 μL of DMSO was added, followed by incubation at 37 °C for 30 min. Absorbance was measured at 560 nm using a microplate reader (Infinite M200 PRO, TECAN, Grödig, Austria).

### 2.3. Flow Cytometry Analysis

SK-Hep-1 cells were plated in 100 mm dishes at a density of 1 × 10^7^ cells per dish and Hep3B cells at 4 × 10^6^ cells per dish. After 24 h, cells were treated with 1 and 3 μM of G1 and 3 μM of the GPER antagonist (G15). Cells exposed to these treatments for varying durations were fixed in PBS containing 70% ethanol and stained with propidium iodide. The cell cycle was then analyzed using a Novocyte2000 flow cytometer (Acea Biosciences, Inc., San Diego, CA, USA).

### 2.4. Western Blotting

SK-Hep-1 cells were plated in 6-well plates at a density of 2 × 10^5^ cells per well and allowed to adhere for 24 h. The cells were then treated with 1 and 3 μM of G1 and 3 μM of G15, which were dissolved in 0.1% DMSO, for 24 h. The control group was treated with 0.1% DMSO alone. In ADAMTS1 and tamoxifen treatment, cells were treated with 10, 50, 100, 200, and 400 ng of recombinant human ADAMTS1 protein (R&D systems, Minneapolis, MN, USA) dissolved in DPBS and 17 μM of tamoxifen (Sigma-Aldrich) dissolved in 0.2% DMSO for 48 h. The control group was treated with 0.2% DMSO alone. Cell lysates were prepared using RIPA buffer (GenDEPOT) containing protease inhibitor (P3100, GenDEPOT) and phosphorylation inhibitors (Roche, Basel, Switzerland). Protein concentrations were measured using a bicinchoninic acid assay, and equal amounts of protein were resolved on 10−15% SDS polyacrylamide gel. After electrophoresis, proteins were transferred onto PVDF membranes (Millipore, Darmstadt, Germany) using a semi-dry blotter (Peqlab, Darmstadt, Germany). Membranes were blocked with 5% skimmed milk (Bio-Rad Inc., Hercules, CA, USA) in Tris-buffered saline with 0.1% Tween 20 (TBST) at 22 °C. Primary antibodies were diluted in TBST and incubated with membranes for 16 h at 4 °C. Incubation of secondary antibody was conducted for 3 h at 22 °C. Protein bands were detected using an enhanced chemiluminescence solution, and images were acquired with a Chemi-doc imaging system (Cytiva Las500, Marlborough, MA, USA). Protein expression images were quantified and analyzed for statistical processing using ImageJ software (ver.1.54k). The following primary antibodies were used: GPER (1:1000, Abcam, Cambridge, UK), p-p53 (1:1000, Santa Cruz Biotechnology, Dallas, TX, USA), cyclin-dependent kinase (CDK) 1 and Cyclin B (1:1000, Cell Signaling, Danvers, MA, USA), extracellular-signal-regulated kinase (ERK) and pERK (1:1000, Invitrogen, Waltham, MA, USA), p53 (1:1000, Millipore), p21 (1:2000, Millipore), ADAMTS1 (1:2000, Rockville, Origene, MD, USA), E-cadherin (1:1000, Cell Signaling), proliferating cell nuclear antigen (PCNA; 1:2000, Cell Signaling), vimentin (1:1000, Cell Signaling), and β-actin (1:5000, Sigma-Aldrich, St. Louis, MO, USA). Secondary antibodies (anti-mouse and anti-rabbit) were used at a 1:3000 dilution.

### 2.5. RNA Sequencing

SK-Hep-1 cells were seeded at a density of 1.5 × 10^6^ cells per dish. After 24 h, the cells were treated with 6 μM of G1 for 24 h. Total RNA was extracted using the TRIzol reagent, and RNA quality was assessed with an Agilent TapeStation 4000 system (Agilent Technologies, Amstelveen, The Netherlands). RNA concentrations were quantified using an ND-2000 spectrophotometer (Thermo Fisher Scientific, Waltham, MA, USA). RNA libraries for control and treated groups were prepared using the QuantSeq 3′ mRNA-Seq Library Prep Kit (Lexogen, Inc., Vienna, Austria) following the manufacturer’s protocol. Briefly, RNA was hybridized with an oligo-dT primer containing an Illumina-compatible adaptor sequence, followed by reverse transcription. After second-strand synthesis and purification, the library was amplified and purified to remove PCR reagents. Single-end 75 bp sequencing was conducted using the NextSeq 550 system (Illumina, Inc., San Diego, CA, USA) (a-16).

### 2.6. Proteomics

SK-Hep-1 cells were seeded at a density of 1.5 × 10^6^ cells per dish and treated with 6 μM G1 for 24 h. Cells were lysed with RIPA buffer (GenDEPOT) containing protease and phosphatase inhibitors. Lysates were sonicated for 30 s (3 s intervals, 50 μm amplitude) using a QSONICA system (Newtown, CT, USA). Proteins were denatured by heating at 95 °C for 10 min and centrifuged at 13,000× *g* for 10 min at 25 °C. Protein digestion was performed using Strap Mini (PROTIFI, Farmingdale, NY, USA), and peptides were labeled with TMT 10 plex (Thermo Scientific, Waltham, MA, USA). The labeled peptides were separated via an Acquity UPLC system (Waters, Milford, MA, USA) and analyzed using an RSLCnano u3000/Orbitrap Exploris 240 mass spectrometer (Thermo Fisher Scientific). Peptide data were processed using MaxQuant v2.0.3.0 (Max-Planck-Institut, Planegg, Germany), and statistical analyses were performed with Perseus software (v1.5.8.0, Max-Planck-Institute).

### 2.7. Protein–Protein Interaction

A search tool for retrieving interacting genes (STRING) database was employed to analyze protein–protein interactions: GPER, MAPK1, CDKN1A, E2F2, BRIP1, CDK1, TUBA1C, TUBB, CDC45, BARK1, TOP2A, CDC25C, UBE2C, FAM83D, NUSAP1, CCNB1, CDC20, EXO1, HELLS, CCNB2, CKS1B, and TP53.

### 2.8. Animal Studies

All animal experiments were approved by the Animal Experiment Ethics Committee (approval number 2022-001-012). Five-week-old male NOD-Prkdcem1Baek Il2rgem1Baek (NSGA) immunodeficient mice were obtained from Jabio Co., Ltd. (Suwon, Republic of Korea) and acclimated for one week in the animal facility at the Duksung Women’s University. Mice were housed under controlled conditions (23 °C, 60% humidity, 12 h light/dark cycle) with ad libitum access to food and water, following the Duksung Women’s University Animal Experiment Ethics Committee guidelines. SK-Hep1 cells (5 × 10^6^ cells/100 μL) were subcutaneously injected into the right hind leg of NRGA mice (G1 growth curve experiment *n* = 10, G1 inhibition metastasis experiment *n* = 12). When the tumor volume reached approximately 100 mm^3^, the mice were randomized into groups and treated as follows: G1: 10 mg/kg by intraperitoneal injection (i.p.), three days a week or vehicle for 6 weeks; tumor volume was measured using a vernier caliper three times per week and calculated using the following equation:

Tumor volume = [Length × (width)^2^]/2

### 2.9. Immunohistochemistry (IHC) and Hematoxylin Staining

Tissue sections (5 μm thick) were mounted on glass slides, washed with PBS, and blocked with PBST containing 1% bovine serum albumin (BSA) for 1 h. Slides were incubated overnight at 4 °C with primary antibodies against p53 and ADAMTS1 (dilution 1:150). After washing, secondary antibodies (anti-mouse) were applied for 2 h at 22 °C, followed by hematoxylin staining. Slides were mounted with a mounting solution and observed under a microscope.

### 2.10. Invasion Assay

Matrigel (Merck, Kenilworth, NJ, USA) was thawed by transferring it from −20 °C to 4 °C for 12 h to achieve a liquid state. The upper chamber of a 24-well transwell plate (SPL, Pocheon, Republic of Korea) with an 8 µm pore size was coated with Matrigel at a concentration of 2 mg/mL in PBS and incubated for 1 h. After solidification, 1 × 10^5^ cells suspended in serum-free medium with 0.1% BSA were added to the upper chamber. SK-Hep-1 cells seeded in the upper chamber were treated with 200 ng of ADAMTS1, 1 and 3 μM of G1, or 3 μM of G15. In control experiments, cells received 0.1% DMSO as the vehicle for G1 treatment and DPBS as the vehicle for ADAMTS1. The bottom chamber contained culture medium supplemented with 10% FBS and 1% penicillin–streptomycin. After 48 h incubation, non-invading cells were removed, and invading cells were fixed, stained with crystal violet, and visualized under a microscope.

### 2.11. Overall Survival Analysis of Differentially Expressed Genes

The OS data of GPER and ADAMTS1 expression were obtained from patients with liver cancer in the Kaplan–Meier plotter database (http://kmplot.com/analysis/, accessed on 31 October 2024). The log-rank *p*-value and hazard ratios with 95% confidence intervals were calculated.

### 2.12. Statistical Analysis

Data are expressed as the mean ± standard deviation. Statistical comparisons between groups were performed using one-way analysis of variance (ANOVA) and the *t*-test in GraphPad Prism 7 (GraphPad Software Inc., San Diego, CA, USA). A *p*-value < 0.05 was considered statistically significant.

## 3. Results

### 3.1. GPER Activation in Liver Cancer Cell Proliferation and Signaling

**Figure 1:**
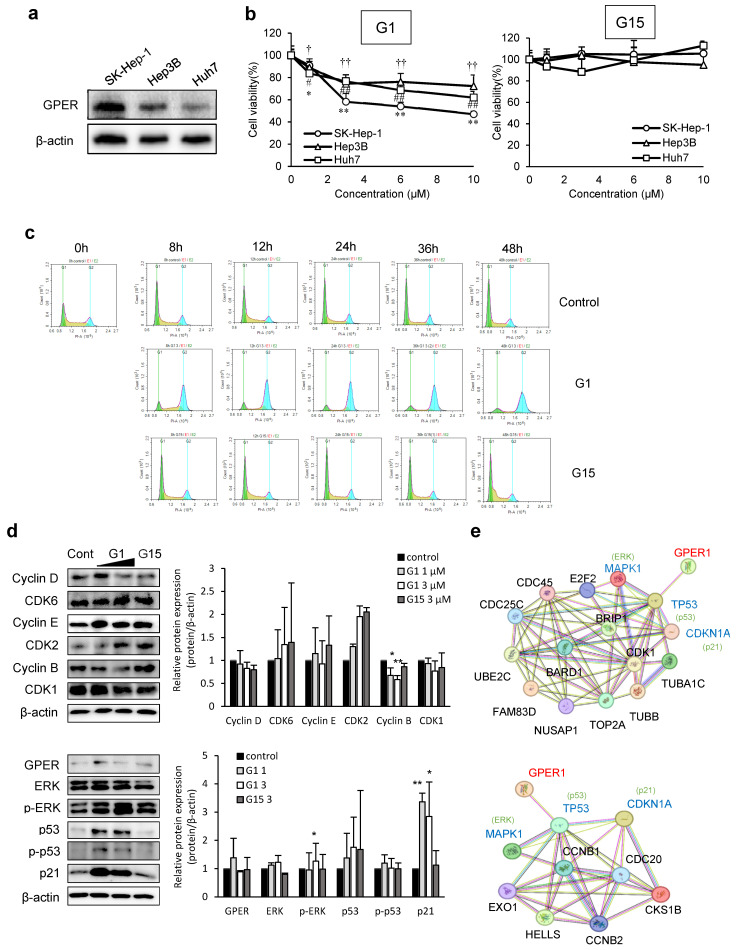
Expression of cell cycle-related and GPER signaling pathway-related proteins. (**a**) Expression levels of endogenous GPER in SK-Hep-1, Hep3B, and Huh7 cells. (**b**) The liver cancer cell lines were treated with G1 (left panel) and G15 (right panel) for 24 h. Cytotoxicity was measured using the MTT assay. Data are presented as the mean ± standard deviation (*n* = 6) * *p* < 0.05, ** *p* < 0.001, # *p* < 0.05, ## *p* < 0.001, † *p* < 0.005, †† *p* < 0.001 (one-way ANOVA with Tukey’s post hoc test). (**c**) SK-Hep-1 cells were treated with 3 µM G1 and G15 for 0, 8, 12, 24, 36, or 48 h. The cell cycles were analyzed via flow cytometry. (**d**) SK-Hep-1 cells were treated with 1, 3 µM G1, and 3 µM G15 for 24 h, and the levels of cell cycle-related and GPER signaling-related proteins were measured via Western blotting. Data are presented as the mean ± SD of three independent experiments. * *p* < 0.05, ** *p* < 0.01 compared to control. (**e**) Correlation analysis of downregulated cell cycle-related genes, proteins, and GPER using STRING, a bioinformatics tool. (Upper panel) Genes derived from cell cycle-related genes under the condition of fold change ≤ 0.36, *p* < 0.005. Interactions with a confidence score of ≥0.4 are shown. Protein–protein interaction enrichment *p*-value < 1.0 × 10^−16^. (Lower panel) Proteins derived from cell cycle-related proteins under the condition of fold change < 0.7, *p* < 0.005. Interactions with a confidence score of ≥0.4 are shown. Protein–protein interaction enrichment *p*-value: 3.36 × 10^−6^. TP53; p53, CDKN1A; p21, MAPK1; ERK. The original images of the Western Blotting figures can be found in Supplementary Figure S1.

GPER expression levels were assessed in Hep3B, Huh7, and SK-Hep-1 cells (Figure 1a), and G1-induced cytotoxicity was dose-dependent (Figure 1b). G1-induced cell cycle arrest at the G2-M phase, whereas G15 had no significant effect on the cell cycle in both SK-Hep-1 cells (Figure 1c). In G1-treated cells, cyclin B expression was significantly suppressed (Figure 1d). We conducted subsequent experiments in SK-Hep-1 cells with high GPER expression. Additionally, downstream signaling components of GPER, such as p-ERK, were highly expressed, with a significant upregulation of p21 and non-significant increase in p53 levels (Figure 1d). To further investigate cell cycle regulatory factors, we performed RNA sequencing and proteomic analysis of the G1-treated SK-Hep-1 cells and identified the differentially expressed genes (DEGs) (Supplementary Tables S1 and S2). Using the STRING database, correlations between downregulated cell cycle regulatory RNAs, proteins, and GPER were analyzed (Figure 1e). Our results suggest that G1 treatment downregulated the key cell cycle regulators at the RNA and protein levels, with GPER facilitating these changes via the p53-mediated pathway. Specifically, our findings indicated that GPER may modulate CDKN1A (p21) and MAPK1 (ERK) expression through p53, suppressing the expression of cell cycle regulators.

### 3.2. Tumor Growth Suppression by GPER Agonists in Liver Cancer Xenograft Models

**Figure 2:**
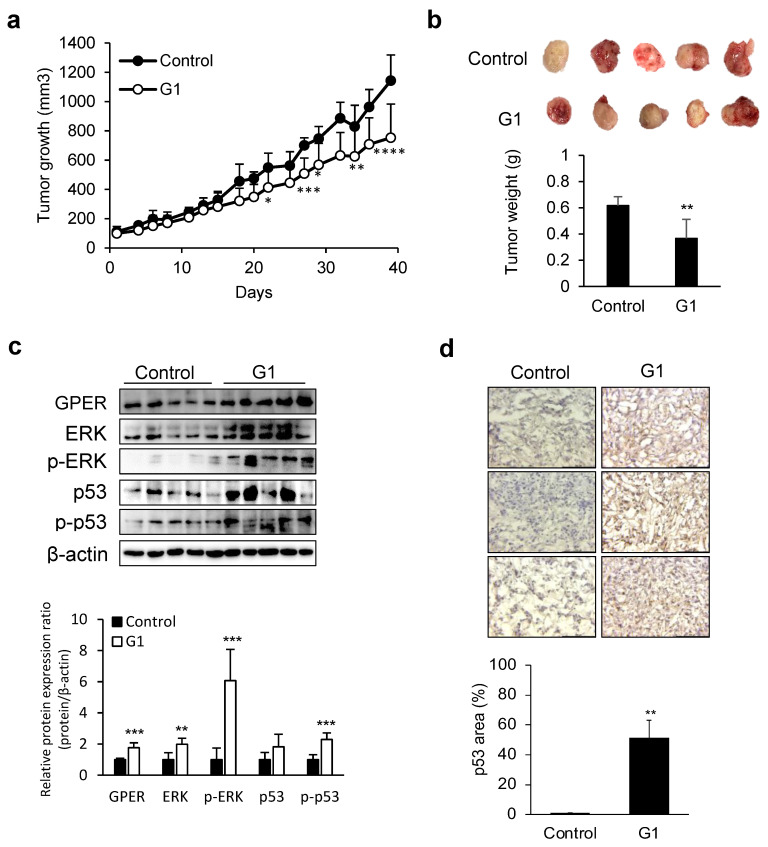
Inhibition of tumor growth by GPER in a liver cancer xenograft model. (**a**) Tumor sizes were measured thrice weekly for 39 days. Data are presented as mean ± standard deviation (*n* = 5) (two-way ANOVA with Sidak’s post hoc test). (**b**) Comparison of tumor tissues between the control and G1-treated groups. Data are presented as mean ± standard deviation (*n* = 5) (two-tailed unpaired *t*-test). (**c**) Confirmation of alterations in GPER signaling after G1 treatment of the SK-Hep-1 cell-derived tumor. The relative protein expression was normalized to β-actin expression using ImageJ. Data are presented as mean ± standard deviation (*n* = 5) (two-tailed unpaired *t*-test). (**d**) Increase in p53 expression levels in SK-Hep-1 cell-derived tumors. The expression levels of p53 in tumor tissues. Images were captured at 40× magnification. The graph shows the area of p53 calculated using ImageJ. Data are presented as mean ± standard deviation (*n* = 3) (two-tailed unpaired *t*-test). * *p* < 0.05, ** *p* < 0.01, *** *p* < 0.001, **** *p* < 0.0001. The original images of the Western Blotting figures can be found in Supplementary Figure S2.

We evaluated the tumor-suppressive effects of G1 in a liver cancer xenograft mouse model. Tumor growth was significantly inhibited in the G1-treated group (Figure 2a). Tumor weight was significantly lower in the G1-treated group than in the control group (Figure 2b). Following treatment, analysis of tumor tissues revealed changes in protein expression consistent with in vitro findings, indicating that GPER, ERK, p-ERK, and p-p53 were significantly upregulated in the G1-treated group (Figure 2c).

IHC analysis demonstrated a substantial increase in p53 expression in G1-treated tumors, emphasizing the role of GPER activation in tumor-suppressive signaling pathways (Figure 2d). These results suggest that GPER is crucial in suppressing liver cancer progression.

### 3.3. ADAMTS1-Mediated Inhibition of EMT and Metastasis

**Figure 3:**
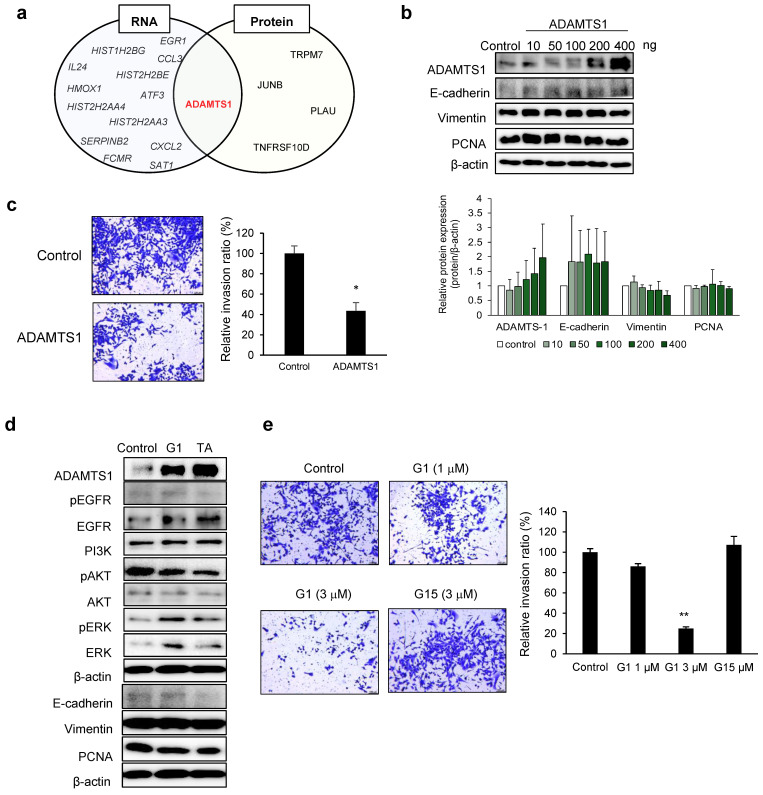
ADAMTS1 and GPER agonists inhibit the invasive ability of SK-Hep-1 cells. (**a**) Genes with RNA expression increased by more than 5.0-fold (*p* < 0.0005) and protein expression increased by more than 1.6-fold (*p* < 0.001). (**b**) ADAMTS1-treated SK-Hep-1 cell lysates obtained after 48 h. The expression of EMT-related proteins was measured through Western blotting. The amount of each protein was calculated compared to β-actin using ImageJ. Data are presented as mean ± standard deviation (*n* = 3). (**c**) An invasion assay was performed with ADAMTS1-treated SK-Hep-1 cells after 48 h. Photos were observed via microscopy (10×). Graph representing the area of the invading cell calculated using ImageJ. Data are presented as mean ± standard deviation (*n* = 3). * *p* < 0.01 (two-tailed unpaired *t*-test). (**d**) The protein expression detected in SK-Hep-1 cells treated with G1 (1 µM) and tamoxifen (TA) (17 µM). Cell lysates from G1 and TA-treated SK-Hep-1 cells were collected after 48 h. (**e**) The invasion was confirmed with G1- and G15-treated SK-Hep-1 cells for 48 h. Photos were observed via microscopy (10×). The graph shows the area of invaded cells calculated using ImageJ. Data are presented as mean ± standard deviation (*n* = 3) ** *p* < 0.01 (one-way ANOVA with Dunnett’s post hoc test). The original images of the Western Blotting figures can be found in Supplementary Figure S3.

Upregulated RNAs and proteins among DEGs were identified in G1-treated cell lines (Supplementary Table S1 and S2). ADAMTS1 was identified as a common molecule at upregulated RNA and protein levels (Figure 3a). Subsequently, we treated the cell lines with ADAMTS1 and performed Western blotting to observe intracellular changes. ADAMTS1 expression increased in a dose-dependent manner (Figure 3b). Furthermore, the epithelial phenotype biomarker, E-cadherin, and the mesenchymal phenotype biomarker, vimentin, showed differences in expression; however, these differences were not significant. Similarly, the proliferation marker, PCNA, showed no significant changes after ADAMTS1 treatment. Finally, we observed that cancer cell invasion was significantly inhibited following ADAMTS1 treatment (Figure 3c), indicating its crucial role in regulating cellular invasion.

We investigated whether ADAMTS1 expression is also increased by other GPER agonists using G1 and tamoxifen. In G1- and tamoxifen-treated cells, ADAMTS1 expression was upregulated, and downstream signaling pathways followed a similar activation pattern (Figure 3d). Thus, ADAMTS1 expression is upregulated through GPER activation. G1 inhibited invasion in a dose-dependent manner compared to that in control cells (Figure 3e).

**Figure 4:**
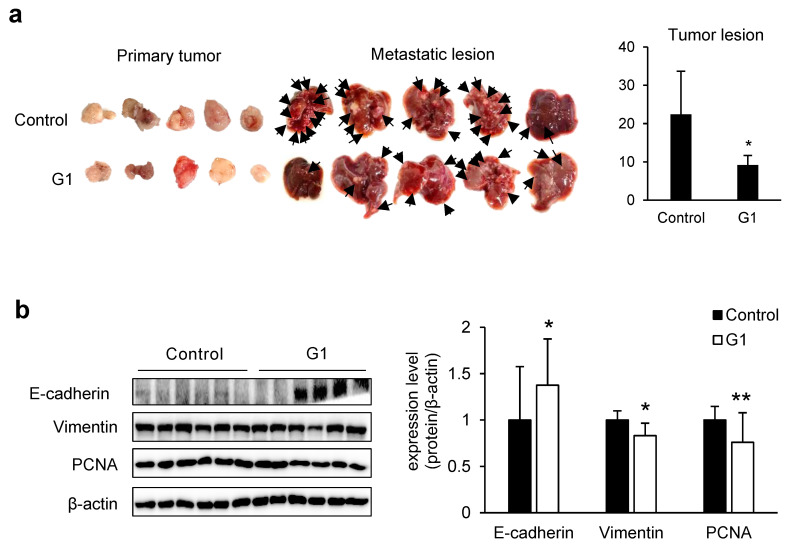

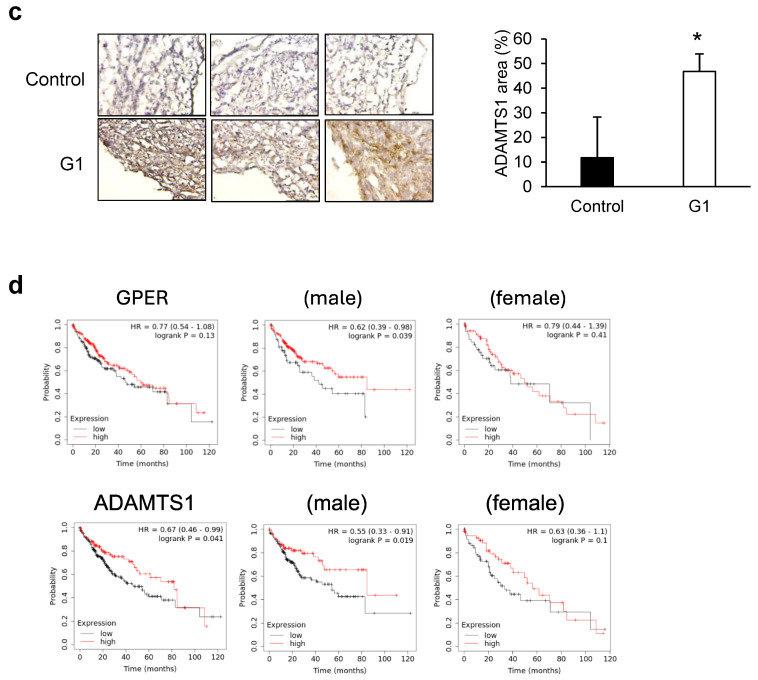
GPER agonist increases ADAMTS1 expression and inhibits metastasis. (**a**) The SK-Hep-1 cell-derived xenograft model and the control group received intraperitoneal administration of G1 at 10 mg/kg thrice weekly. Tumor size and liver metastases were compared between the G1 and control groups (arrow: metastatic lesion). Data are presented as mean ± standard deviation (*n* = 5) * *p* < 0.05 (two-tailed unpaired *t*-test). (**b**) ADAMTS1, E-cadherin, Vimentin, and PCNA levels were measured in the transplanted subcutaneous tumor. The ratio of these protein levels to β-actin was calculated using ImageJ. Data are presented as mean ± standard deviation (*n* = 6) * *p* < 0.05, ** *p* < 0.01 (two-tailed unpaired *t*-test). (**c**) Immunohistochemical staining for ADAMTS1 in the subcutaneously implanted tumor. The area of ADAMTS1 protein levels was calculated using ImageJ. Data are presented as mean ± standard deviation (*n* = 3) * *p* < 0.05 (two-tailed unpaired *t*-test). (**d**) The overall survival rates of liver cancer patients were compared with GPER and ADAMTS1 expression levels in men and women. In the KM plotter database, the gene IDs of GPER and ADAMTS1 were 2852 and 9510, respectively. HR, hazard ratio. Data were analyzed using the optimal threshold as the cut-off. The original images of the Western Blotting figures can be found in Supplementary Figure S4.

To evaluate the anti-metastatic effects of G1 in vivo, we used a metastasis model that permits simultaneous observation of primary and metastatic tumors [17]. NSGA mice were administered SK-Hep-1 cells subcutaneously to induce spontaneous metastasis. Thereafter, G1 was administered intraperitoneally to evaluate its effects on the progression of metastasis. After treatment, the G1-treated group significantly reduced liver metastatic nodule formation (Figure 4a). Western blot analysis of tumor tissues revealed increased E-cadherin expression, decreased vimentin expression, and reduced levels of the PCNA (Figure 4b), indicating that G1 can modulate cell phenotype and suppress cell proliferation. IHC analysis also showed a significant increase in ADAMTS1 expression in peripheral regions of tumors in the G1-treated group (Figure 4c).

In the xenograft model, the reduction in liver metastatic nodules and increased ADAMTS1 expression in tumors in the G1-treated group aligned with clinical data. Thus, higher expression of GPER and ADAMTS1, especially in men with liver cancer, was associated with better OS (Figure 4d). These findings suggest that GPER and ADAMTS1 upregulation may facilitate the inhibition of liver cancer metastasis and improve clinical outcomes.

## 4. Discussion

Although research on liver cancer has progressed steadily, tumor proliferation and metastasis negatively affect patient outcomes. GPER signaling pathways can exhibit pro-tumor and anti-tumor effects. In endometrial carcinoma and ovarian cancer, GPER over-expression was associated with poor prognosis [18,19]. In triple-negative breast cancer, high GPER levels increased rates of death, local relapse, and distant metastasis in pre-menopausal patients [20]. However, the role of GPER in non-reproductive cancers, such as liver cancer, as opposed to breast or reproductive cancers, remains unclear.

In liver cancer, GPER activation has been associated with anti-tumor effects via ERK signaling [3], whereas other studies have reported pro-tumorigenic roles through the PI3K/mTOR pathway [21]. Moreover, GPER knockout has been shown to accelerate liver tumor formation and promote the production of inflammatory factors, leading to adverse outcomes [8]. These findings suggest that GPER plays a role in liver cancer; however, its exact function in tumor promotion or suppression is yet to be further confirmed. Therefore, further research is required to elucidate the precise role of GPER in liver cancer and assess its potential as a therapeutic target.

In this study, we confirmed that GPER activation may inhibit liver cancer progression. GPER agonists regulated the cell cycle, increased p53 expression, and upregulated ADAMTS1 expression in vivo and in vitro, suppressing liver cancer progression. Our findings suggest that GPER is a potential therapeutic target for liver cancer. We found that G1 induced G2-M phase arrest in SK-Hep-1 cells via GPER and downregulated cyclin B expression. Furthermore, G1 treatment also induced G2-M phase arrest in Hep3B cells, supporting that the effect is not limited to SK-Hep-1 cells (Supplementary Figure S5a). This aligns with the findings of previous studies, which showed that G1 also reduced cyclin B levels in breast and prostate cancer cells [21,22,23]. G2-M phase arrest induced by GPER activation has been observed in glioblastoma, ovarian, and breast cancer cells [21,24,25,26].

We demonstrated that G1 significantly upregulated p21, p53, and ERK expression. G1 significantly increased p53 and ERK expression while effectively inhibiting tumor growth in a liver cancer xenograft model. ERK activation is induced by G1 in prostate, breast, and liver cancer cells, with sustained ERK activation reportedly conveying anti-proliferative signals [3,23,27,28]. p53 upregulation has also been observed in G1-treated breast cancer cell lines [21,22]. In human fibroblasts, p21 regulates G1/S and G2/M phases, contributing to cell cycle arrest [29]. Cyclin B downregulation by p21 is associated with G2/M phase regulation [30]. Our data confirmed that G1 increased p21 expression and decreased cyclin B expression, leading to G2/M phase arrest. Activation of ERK and upregulation of p53 also facilitated tumor growth inhibition. These results suggest that GPER activation can suppress liver cancer progression. This aligns with previous studies demonstrating the anti-tumor effects of GPER across various cancer types [7,8,25].

Notably, our study identified ADAMTS1 expression following G1 treatment. We confirmed that ADAMTS1 was significantly upregulated through RNA sequencing and proteomic analysis, indicating a correlation between ADAMTS1 and GPER. ADAMTS1 is closely associated with cancer metastasis through its role in ECM remodeling by degrading substrate proteins, such as collagen, and sequestering vascular endothelial growth factor (VEGF), an angiogenesis inhibitor [31,32]. Our data showed that ADAMTS1 treatment significantly suppressed the invasion ability of SK-Hep-1 cells without cytotoxicity (Supplementary Figure S5b). This is consistent with previous studies, which showed that ADAMTS1 overexpression in ovarian cancer cells also inhibited cell migration and invasion [33]. Additionally, we also observed that G1 treatment may partially reduce the cell viability, effectively suppressing invasion, with molecular analysis showing increased levels of ADAMTS1 and EGFR and decreased levels of pAKT. In contrast, ADAMTS1 overexpression promoted lymph node metastasis through the upregulation of EGFR, pEGFR, and pAKT in oral squamous cell carcinoma [12]. These discrepancies suggest that the function of ADAMTS1 may change due to GPER activation, highlighting the potential variability of its role depending on its regulatory context.

In our spontaneous metastasis model, we evaluated the efficacy of G1. G1 treatment suppressed metastasis not only to the liver but also to the kidneys (Figure 4a and Supplementary Figure S5c). We found that it significantly delayed metastatic progression by suppressing tumor proliferation in primary tumor tissue while exhibiting increased E-cadherin and decreased vimentin expression. This observation is consistent with the anti-metastatic effects of GPER activation, which have also been reported in breast and pancreatic cancers [34,35]. Notably, cell division and migration are most active in tumor margins. Our study revealed a significant increase in ADAMTS1 expression, particularly at the tumor margins, following G1 treatment [36]. ADAMTS1 suppresses tumor growth in human fibrosarcoma and prostate cancer cells by regulating VEGF and fibroblast growth factor 2, thereby inhibiting angiogenesis [37,38,39].

Overall, these findings suggest that GPER activation through G1 influences ADAMTS1 expression, potentially regulating the growth and metastasis of liver cancer. However, ADAMTS1 promotes tumor growth and invasion, particularly in the liver; it has been linked to the exacerbation of liver fibrosis and the promotion of hepatocellular carcinoma [40,41]. Thus, further investigations are required to fully elucidate its role in liver cancer. Additionally, the clinical relevance of GPER and ADAMTS1 is highlighted by their association with higher expression levels and improved OS in men with liver cancer. Our results have limitations in fully elucidating the functional significance of the GPER-ADAMTS1 pathway. A previous report showed that G1-induced GPER upregulation could sensitize cellular response to further GPER activation via a positive feedback loop in the hippocampus [42]. Given that GPER activation also increases ADAMTS1 expression, this feedback mechanism may amplify ADAMTS1 activity. The detailed regulatory mechanism will be investigated in further studies. Our findings suggest that regulating ADAMTS1 via GPER activation is a potential therapeutic target in liver cancer, particularly inhibiting metastasis and improving patient outcomes.

## 5. Conclusions

Our study highlights the therapeutic potential of GPER agonists such as G1 in suppressing liver cancer progression and metastasis. Future studies should focus on elucidating the molecular mechanisms by which GPER regulates ADAMTS1 and its downstream effects on metastasis.

## Data Availability

All datasets generated for this study are included in this article and its Supplementary Data Files.

## Supplementary Materials

The following supporting information can be downloaded at https://www.mdpi.com/article/10.3390/cancers17162623/s1. Supplementary Table S1. Differentially expressed genes. Supplementary Table S2. Differentially expressed genes. Supplementary Figures S1–S4. Western Blotting figures. Supplementary Figure S5. Effects of GPER agonists and ADAMTS1 on cell cycle progression and cytotoxicity in liver cancer cells.

## Abbreviations

The following abbreviations are used in this manuscript:

ANOVA	analysis of variance
BSA	bovine serum albumin
CDK	cyclin-dependent kinase
DEG	differentially expressed gene
ECM	extracellular matrix
EMT	epithelial–mesenchymal transition
FBS	fetal bovine serum
GPER	G protein-coupled estrogen receptor
IHC	immunohistochemistry
OS	overall survival
PCNA	proliferating cell nuclear antigen
TA	tamoxifen
VEGF	vascular endothelial growth factor
