# Supplementary material for: Activation of G Protein-Coupled Estrogen Receptor Induces p53 and ADAMTS1 to Inhibit Tumor Growth and Suppress Liver Cancer Metastasis
Source: Cancers (Basel). 2025 Aug 11;17(16):2623. doi: 10.3390/cancers17162623 (PMC12384126; doi:10.3390/cancers17162623)
Supplement: Supplementary file 1 [file cancers-17-02623-s001.zip › cancers-3792122-supplementary.pdf]

Supplementary Table S1. Differentially expressed genes

Gene symbol	Log (Fold change)	*p*-value
UBE2C	0.228	0.0000
TOP2A	0.242	0.0001
CDK1	0.266	0.0001
CDC25C	0.278	0.0037
E2F2	0.294	0.0015
NUSAP1	0.313	0.0000
CDC45	0.324	0.0015
FAM83D	0.335	0.0000
BRIP1	0.350	0.0000
TUBB	0.354	0.0004
BARD1	0.359	0.0000
TUBA1C	0.360	0.0001

Supplementary Table S2. Differentially expressed genes

Gene symbol	Log (Fold change)	*p*-value
CKS1B	0.536	0.0003
CDC20	0.604	0.0001
CCNB1	0.622	0.0001
EXO1	0.682	0.0000
HELLS	0.685	0.0026
CCNB2;CCNB2V	0.690	0.0003

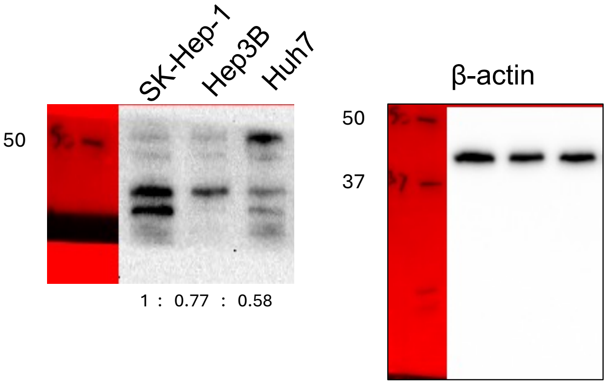

Supplementary Figure S1

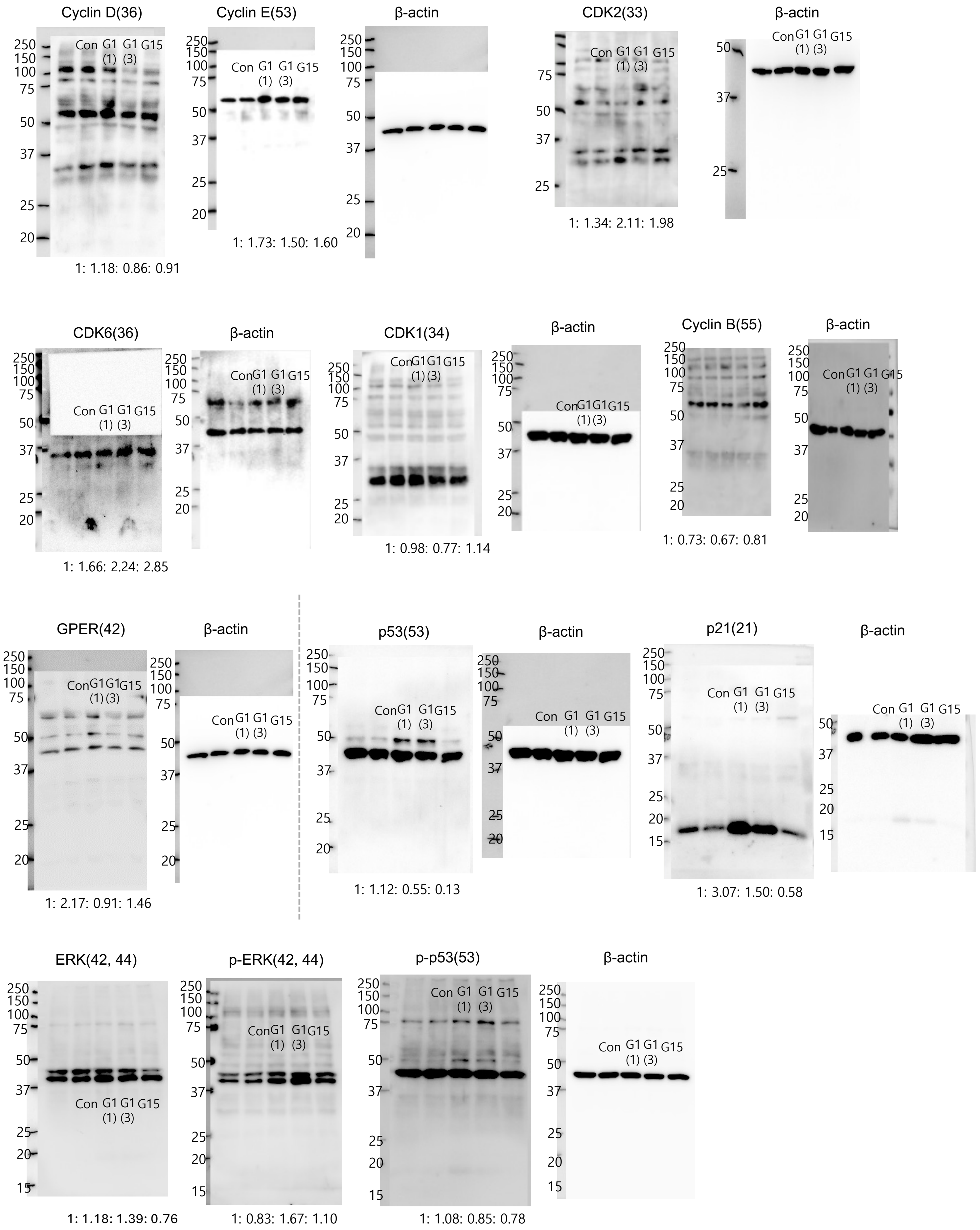

Supplementary Figure S1

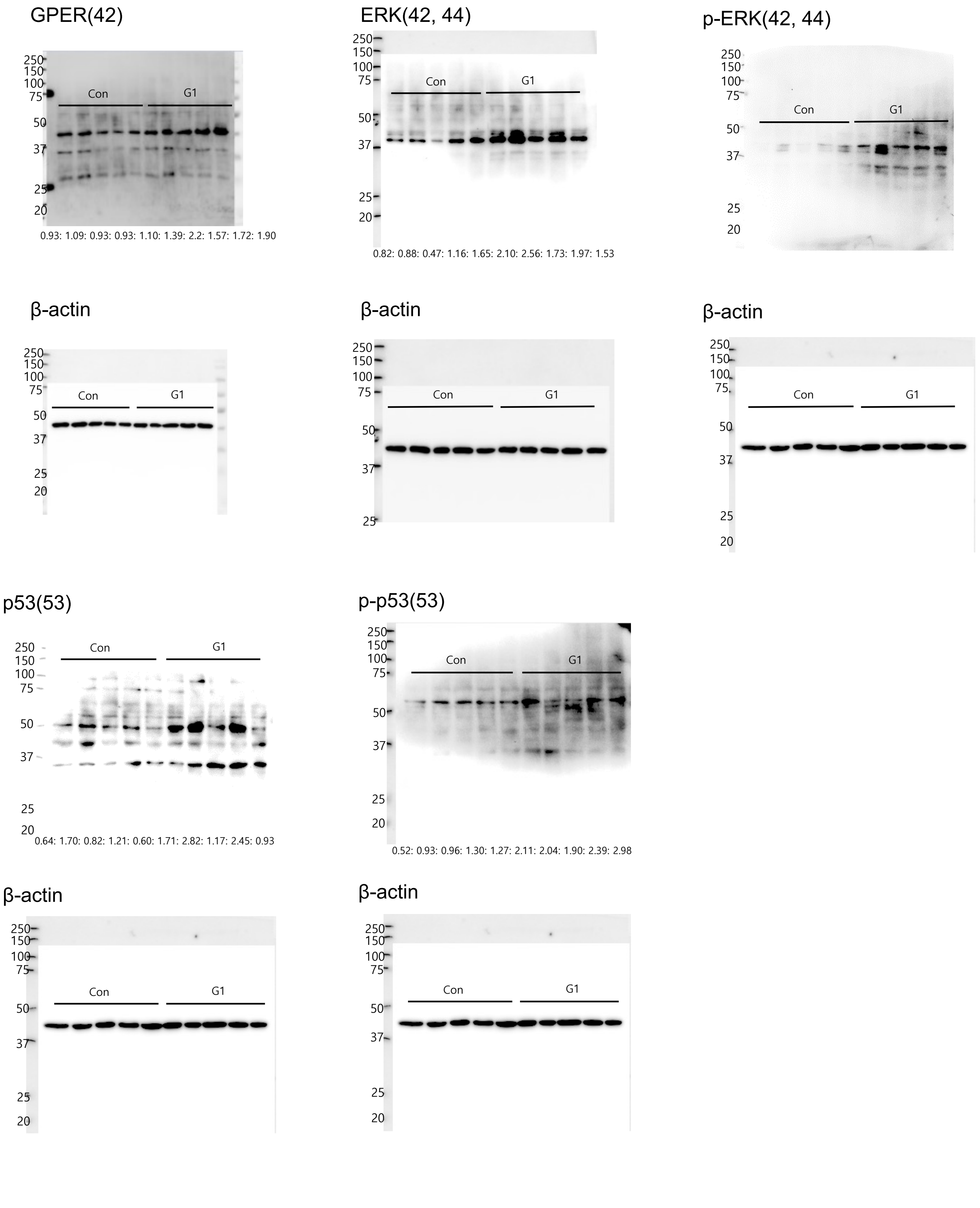

Supplementary Figure S2

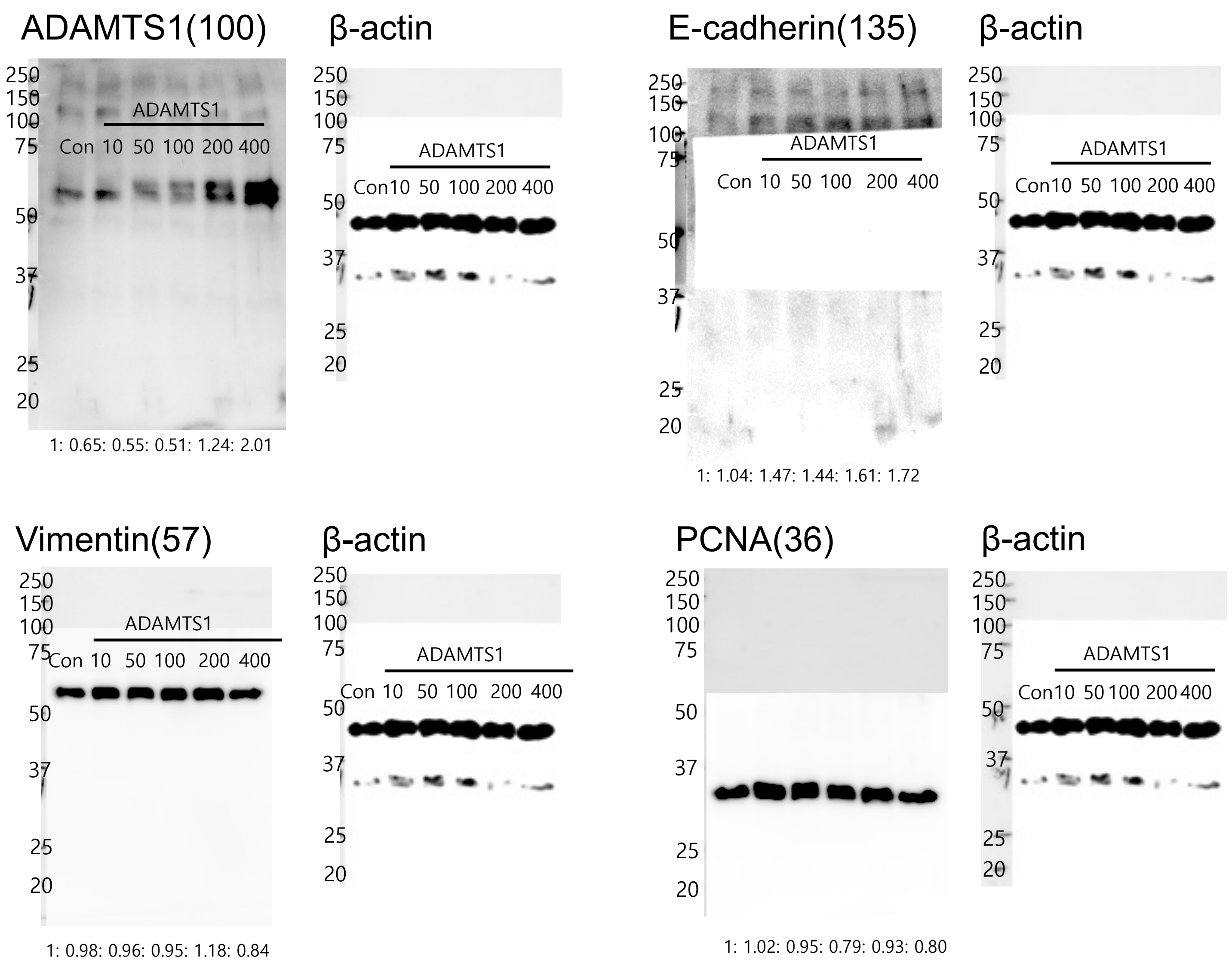

Supplementary Figure S3

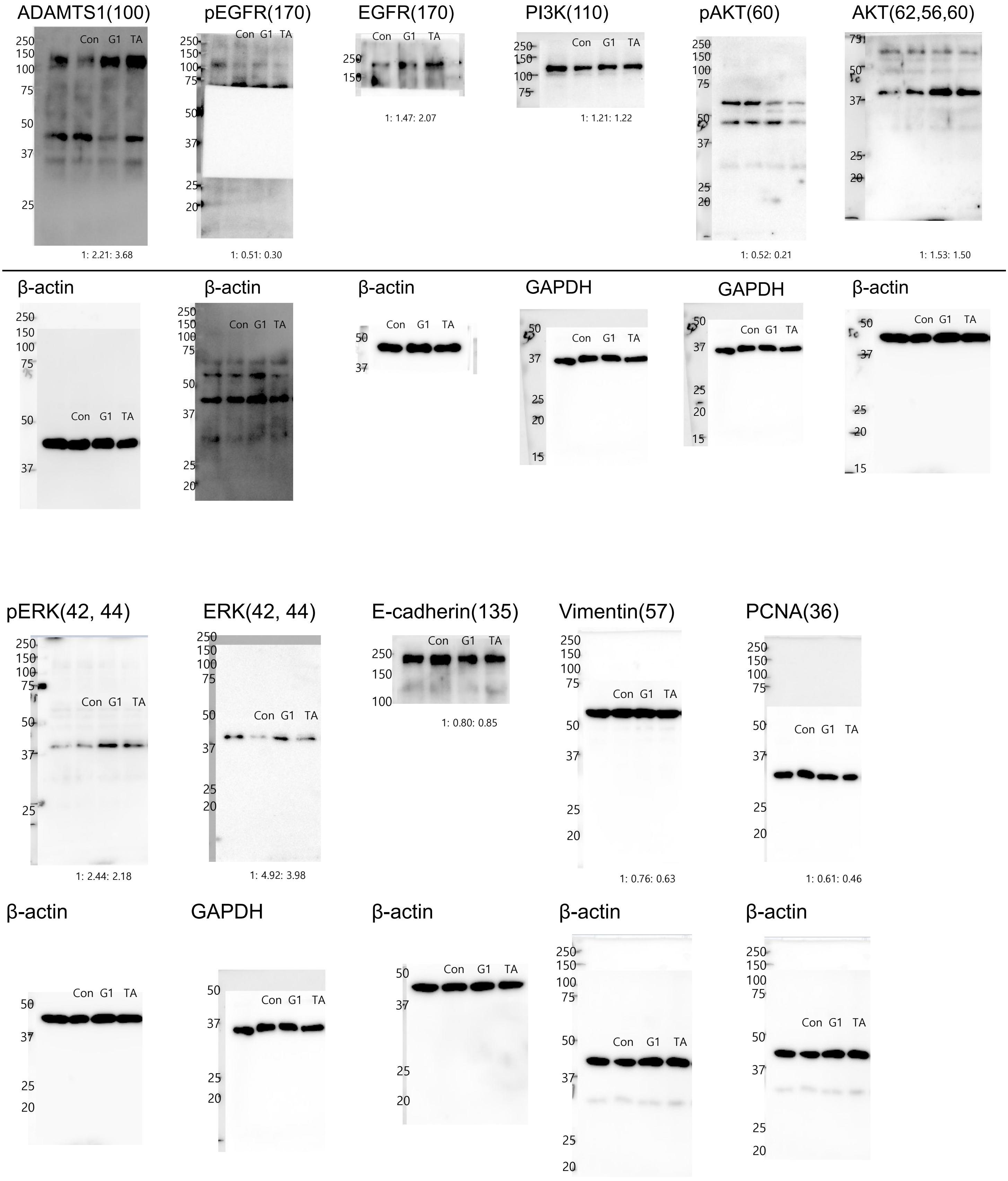

Supplementary Figure S3

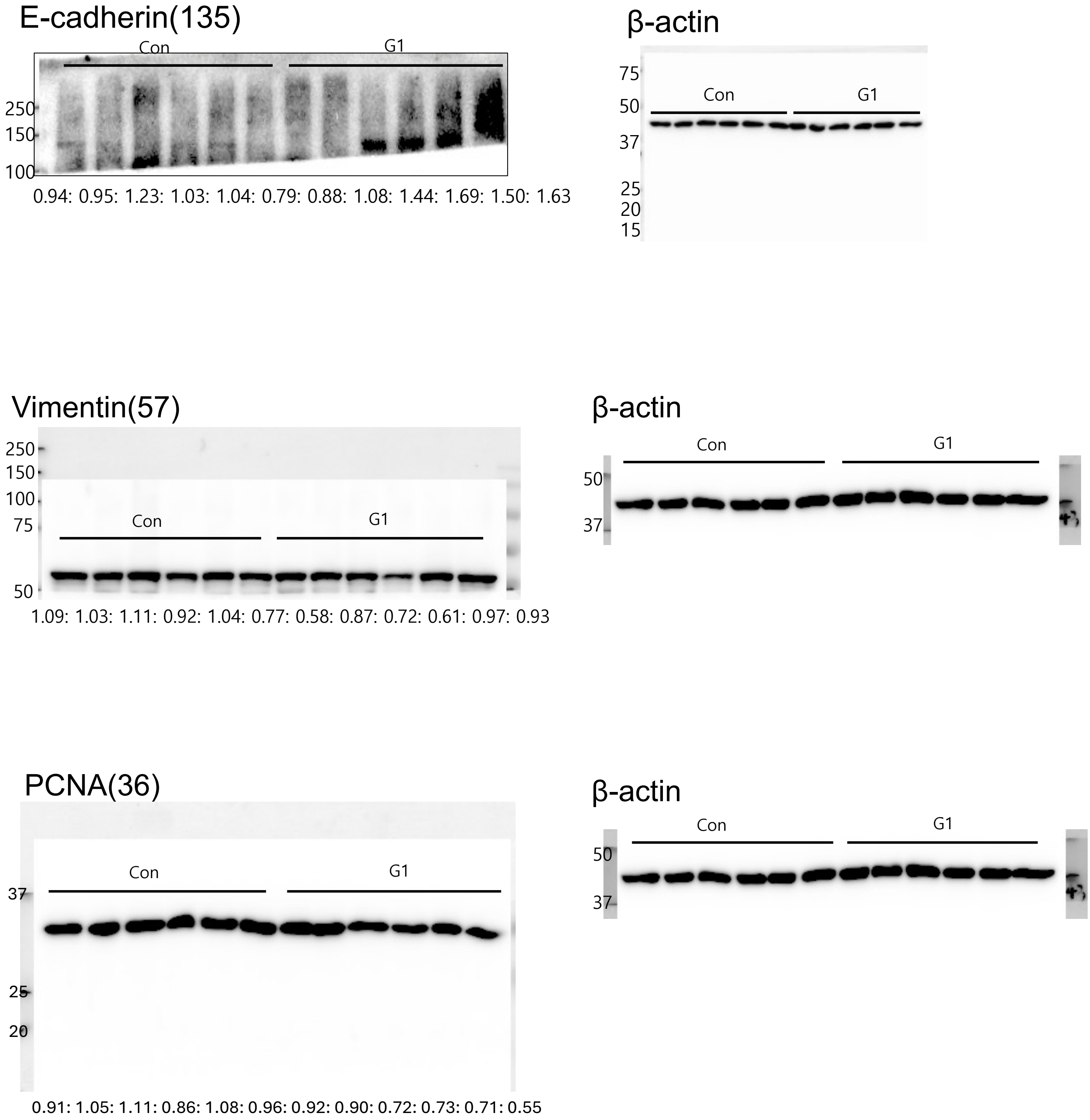

Supplementary Figure S4

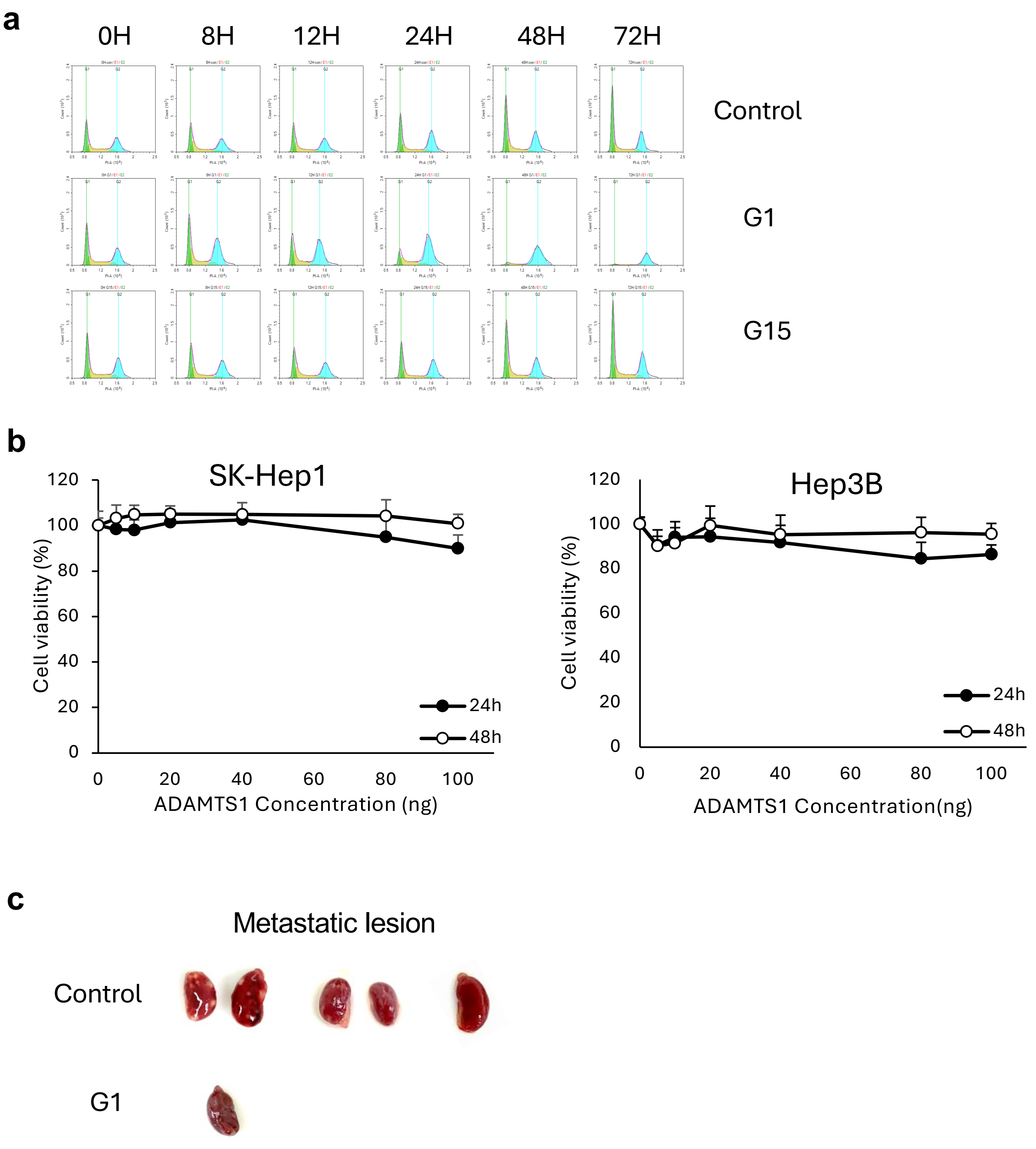

Supplementary Figure 5. Effects of GPER agonists and ADAMTS1 on cell cycle progression and cytotoxicity in liver cancer cells.

(**a**) Hep3B cells were treated with 3 μM G1 and G15 for 0, 8, 12, 24, 48, or 72 h. Cell cycle distribution was analyzed using flow cytometry.

(**b**) SK-Hep-1 (left panel) and Hep3B (right panel) cells were treated with ADAMTS1 (5, 10, 20, 40, 80, or 100 ng) or DPBS (control) for 24 or 48 h. Cytotoxicity was assessed using the MTT assay. (**c**) Representative image of metastatic kidney tissue from a mouse treated with G1.
